# Impact of a Mandatory Two-Year Job Rotation System on Turnover Intention Among Registered Nurses: A Case Study at a Medical Center in Taiwan

**DOI:** 10.1097/jnr.0000000000000758

**Published:** 2026-06-29

**Authors:** Hsin-Ling TAI, Seng-Su TSANG, Shu-Fen CHEN, Jiu-Yun TIAN

**Affiliations:** 1 Department of Business Administration, National Taiwan University of Science and Technology, Taipei, Taiwan; 2 Department of Nursing, Taipei Veterans General Hospital, Taipei, Taiwan

**Keywords:** affect valuation theory, emotional resilience, job stress, nursing adaptation, nurse retention

## Abstract

**Background:**

Taipei Veterans General Hospital in Taiwan experiences significantly high turnover rates among its workforce of approximately 3,300 nurses.

**Purpose:**

This study, conducted as a component of a talent revitalization strategy, was designed to investigate the impact of a mandatory two-year job rotation system on turnover intention, job stress, and emotional well-being among nurses at a major medical center in Taiwan.

**Methods:**

A cross-sectional design was used on a convenience sample of 246 nurses. Factors measured included job stress, job satisfaction, stress levels, emotional resilience, and turnover intention. Partial least squares structural equation modeling (PLS-SEM) and affect valuation theory (AVT) were employed to assess the relationships between variables.

**Results:**

The findings showed that, while job rotation promoted professional versatility, it also introduced significant psychological stressors that may increase turnover risk. In detail, a gap was identified between the actual emotional responses of nurses and their ideal affective states that highlights a need for better emotional support systems and flexible rotation policies.

**Conclusions:**

The results of this research indicate a need to enhance career development opportunities, introduce voluntary rotation options, and implement comprehensive training and mentorship programs to better align the current rotation system with individual career goals.

## Introduction

Retaining skilled nursing staff is an ongoing challenge in the healthcare sector, with high turnover rates impacting both patient care quality and operational costs (Bae, 2022; Xu et al., 2023). At Taipei Veterans General Hospital (TVGH), turnover is especially high among its approximately 3,300 nursing staff. A primary contributor to this workforce issue was posited to be the mandatory two-year job rotation system in place at TVGH, which affects 250–300 postgraduate year (PGY) nursing trainees annually. The excessively high (50%) turnover rate at TVGH for nurses in their first three years of employment has been linked to this rotation system.

Job rotation is a strategic management tool used widely across industries to reduce monotony and promote multiskill development (Lei et al., 2022). In nursing, job rotation aims to support professional growth by improving clinical competencies and increasing awareness of specialized care areas (Platis et al., 2022). However, frequent transitions also present challenges, requiring nurses to rapidly adapt to new environments, which often increases levels of stress and job dissatisfaction (Kim & Kim, 2021; Senek et al., 2020). The dual effect of professional growth accompanied by higher turnover risk warrants further investigation. Therefore, this cross-sectional study was conducted to investigate the impact of a two-year job rotation system on nurse turnover intention at TVGH by exploring the relationships among job satisfaction, emotional resilience, and job stress.

### Psychological Effects of Job Rotation on Nursing

Job rotation, a common practice in nursing, is a strategy aimed at addressing the diverse nature of clinical tasks (Platis et al., 2022). Prior studies have shown inter-departmental rotations to enhance professional knowledge, technical skills, and teamwork abilities in nurses (Bracarense et al., 2022), fostering a more comprehensive understanding of healthcare operations and strengthening conflict-resolution skills. However, this practice also poses challenges in terms of increasing work-related stress and anxiety (Gurung et al., 2020), particularly in cases where nurses are required to acquire new skills and adapt to unfamiliar environments (Alfuqaha et al., 2021; Lei et al., 2022). Because of the potential adverse effects of mandatory job rotations on performance and turnover intention, the provision of psychological support (Kim & Han, 2020), adequate training, and clear communication has been recommended to help nurses navigate the process (Zhong et al., 2024).

### Impact of Job Rotation on Work-Related Stress, Satisfaction, and Adaptability

Job stress is a persistent issue in healthcare. Resilience, as defined by Smith et al. (2008), plays a key role in mitigating this stress by helping individuals recover from stressors, maintain emotional stability, and sustain a positive work attitude (Cha et al., 2022; Jeong & Cho, 2020). The COVID-19 pandemic provided a backdrop for studies such as Moisoglou et al. (2024), who used the Brief Resilience Scale (BRS) to examine the link between resilience and job burnout among nurses. Their findings highlighted the protective role of resilience and social support during crises and job burnout. Furthermore, job rotation stress has been shown to affect nursing work performance among clinical nurses, with resilience moderating this relationship (Jeong et al., 2023).

Emotional resilience significantly influences the adaptability of nurses to job rotations. Ranjbar and Gorji (2018) found that high adaptability and resilience help nurses better manage rotation-related stress, which reduces turnover risk. Earlier, Connor and Davidson (2003) identified resilience as a crucial psychological factor in minimizing turnover and enhancing job satisfaction. Finally, the longitudinal investigation by Dong et al. (2023) of the interplay between career adaptability, professional commitment, and anxiety highlighted the value of professional support in fostering commitment and easing emotional strain during job rotations.

### Theoretical Framework

This study is grounded in affect valuation theory (AVT), as described in a study by Tsai (2007), and the stress coping theory described in a study by Lazarus (1993). Using cultural and temperamental factors, AVT integrates a desired emotional state with contemporary models of affect and emotion. While the dual approach taken in stress coping theory conceptualizes coping as a personality characteristic, AVT conceptualizes it as a dynamic process that evolves over time and is shaped by the adaptational context in which it is generated.

The objectives of this research are to: (1) examine the impact of job rotation on job stress among newly recruited nurses, (2) investigate the role of resilience in mitigating the adverse effects of job rotation, (3) assess the relationship between job satisfaction and turnover intention, and (4) analyze how emotional responses influence job performance during the rotation process.

## Methods

### Research Design

A cross-sectional survey was conducted at the Taipei Veterans General Hospital (TVGH) between April 12, 2023 and March 14, 2024, during which 260 questionnaires were distributed using a convenience sampling method. The inclusion criteria were defined as full-time PGY nurses with seven months to three years of rotation experience. Fourteen nurses declined to participate, resulting in 246 valid responses (94.6%).

### Measures

The PGY Nursing Rotation Questionnaire was used in this study to assess job satisfaction, stress, resilience, and emotional well-being. The instrument has been previously validated by expert reviewers and incorporates a balance of positively and negatively worded items to minimize social desirability bias. In this study, emotional responses were evaluated separately during work and rest periods using the Chinese version of the AVI Emotion Scale to track emotional fluctuations across the rotation cycle. This questionnaire demonstrated strong reliability and validity, with a high-reliability score (Cronbach’s *α* = .91) and a good model fit (GFI) = .973.

The Nursing Job Rotation Stress Scale was used in this study to measure variables such as individual strengths, knowledge, and skill enrichment in other fields. All scale items are measured using a five-point Likert Scale (1=*strongly disagree*, 5=*strongly agree*). The Cronbach’s *α* for this scale was .87, and subscale reliability scores ranged from .80 to .84, confirming its reliability and validity in assessing job rotation stress in nurses.

The BRS was used to evaluate the capacity of nurses to recover from (i.e., resilience to) job-related stress. The BRS comprises six items, with three positively and three negatively worded. The Cronbach’s *α* of the BRS was previously calculated as .91, and model fit is good (GFI = .973).

### Ethical Considerations

All of the participants provided written informed consent. The institutional review board (2023-02-001ACF) approved this study.

### Data Collection

Data collection was conducted by the research team and nursing staff. The survey was conducted using physical paper-based questionnaires, which were completed by the participants, sealed, and then returned to the principal investigator’s department (the Hemodialysis Unit), where they were securely stored.

### Statistical Analysis

Descriptive and multivariate statistical techniques were used to analyze data to examine the relationships between variables and assess the impact of the rotation system. Descriptive statistics were used to summarize the sample and identify underlying patterns in the data. Partial least squares–structural equation modeling (PLS-SEM) was applied to evaluate the relationships among multiple latent constructs influencing turnover intention. PLS-SEM was selected for its ability to handle complex models and explain variance in dependent variables. The analysis focused on the following 11 constructs: work adjustment (WA), resilience during rotation (RR), job stress during a rotation (JS), turnover intention (TI), perceptions of the rotation process (RP), job performance post-rotation (JP); emotional response (ER), willingness to rotate again (RW), job role behavior (RB), interpersonal communication (IC), and impact on daily life (DL).

### Partial Least Squares–Structural Equation Modeling Path Analysis

The PLS analysis involved verifying the reliability and validity of the measurement instruments. Composite reliability (CR) and average variance extracted (AVE) were calculated for all of the constructs. The significance of hypothesized paths was tested using the bootstrap resampling method. Path coefficients and the variance explained (*R* ^2^) were examined to evaluate the explanatory power of the model.

The focus of the PLS-SEM analysis was on examining how various rotation-system-related factors influenced turnover intention. The key components of this analysis included:

Path coefficients: The strength and direction of the relationships between latent variables were assessed using path coefficients, providing insights into which factors significantly affect turnover intention.

Significance testing: *t*-values and *p*-values were used to determine the statistical significance of each relationship, with *t*-value >1.96 was considered statistically significant at the 95% confidence level, and *p*-values <.05 indicated statistical significance.

Model interpretation: The direct and indirect effects of the rotation system on turnover intention among the participants were identified.

### Analytical Focus

The PLS-SEM model was used to evaluate the following key relationships:

Work adjustment and job stress: How the rotation process affects stress levels and job adaptability.

Rotation perception and job satisfaction: How perceptions of the rotation system influence overall job satisfaction.

ER and TI: The connection between emotional experiences during the rotation process and subsequent turnover intention.

IC and job performance: How communication skills developed during the rotation process impact job performance.

Missing data were addressed using mean substitution to ensure consistency and preserve analysis stability, with the ideal mean and ideal median representing the expected frequencies of experiencing ideal emotional states.

## Results

### Participants

A significant majority (197, 80.1%) of the 246 participants were females. The average age was 27 (*SD* = 0.6) years, and average work experience was 48.33 (*SD* = 8.55) months. Two of the participants held a master’s degree, and two held an associate’s degree, with the remainder holding degrees from university-level nursing colleges or departments. In terms of nursing competence, 100 were classified at the N2 level, 31 at N1, and the remainder at N. Thirteen were married, and three reported having children.

### Overview of the Path Coefficients

**Figure 1:**
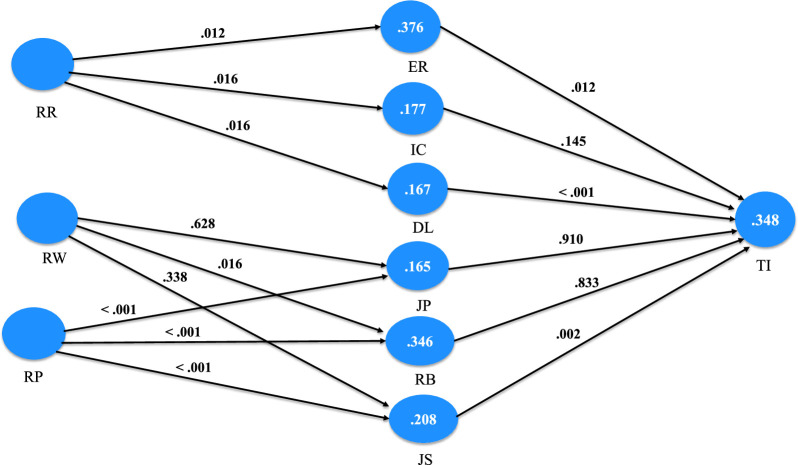
PLS-SEM Structural Model With Path Coefficients *Note.* Values on the arrows are p values; values inside endogenous constructs are *R* *^2^*; RR, RW, and RP are exogenous constructs and therefore do not display *R* *^2^* values. *R* *^2^* values are presented only for endogenous constructs in the PLS-SEM model. PLS-SEM = partial least squares structural equation modeling; RR = resilience during the rotation; RW = willingness to rotate again; RP = perceptions of the rotation process; ER = emotional response; IC = interpersonal communication; DL = impact on daily life; JP = job performance post-rotation; RB = job role behaviour; JS = job satisfaction during rotation; TI = turnover intention.

In PLS-SEM, path coefficients function similarly to regression weights and are used to indicate both the strength and direction of relationships between latent variables (Figure 1). These coefficients were used to explore how various factors within the job rotation system influence Tis. The following key relationships were identified:

- DL → TI (direct lineage to turnover intention): The path coefficient was calculated as 0.398, indicating a moderately positive relationship between the clarity of career pathways and turnover intention. Higher scores indicate a greater likelihood of turnover when career trajectories are perceived as unclear, highlighting the need for greater transparency in career development structures within nursing management.
- ER → TI (emotional response to turnover intention): The path coefficient was calculated as 0.239, indicating a significant impact of emotional responses on turnover intention. This suggests negative emotional experiences during job rotations correlate positively with increased desire to leave, underlining the necessity of implementing emotional support mechanisms during the rotation process.
- JS → TI (job satisfaction to turnover intention): The path coefficient was calculated as −0.264, indicating a significant negative association. In light of the inverse relationship of JS with turnover intention, JS wields a significant influence on nurse retention, particularly in settings with frequent job rotations.

### Significance Testing: *t*-values and *p*-values

Statistical significance was evaluated using *t*-values and *p-*values to assess the reliability of the estimated path coefficients.

DL → TI:

- *t*-value: 5.54 (exceeding the threshold of 1.96), indicating a statistically significant relationship at the 95% confidence level.
- *p*-value:<.001, affirms the robustness of the result.
- Implication: Career pathway clarity significantly influences TI, with unclear job trajectories elevating the risk of turnover. This finding underlines the importance of implementing transparent career development frameworks.

ER → TI:

- *t*-value: 2.52 (above the critical threshold), confirming statistical significance.
- *p*-value: .0116, supporting the positive relationship between emotional responses and TI.
- Implication: Negative emotional factors such as job-related stress and dissatisfaction with work-life balance are essential in determining the likelihood of leaving. This finding underlines the importance of implementing effective emotional support systems.

JS → TI:

- *t*-value: 3.14, confirming statistical significance.
- *p*-value: .0017, validating that JS plays a protective role against turnover.
- Implication: Increasing JS using targeted strategies can significantly reduce turnover rates, especially in high-pressure settings such as nursing.

### Nonsignificant Relationships

Not all of the relationships hypothesized in this study demonstrated statistical significance:

IC → TI (interpersonal communication to turnover intention):

- *t*-value: 1.46 (below the critical threshold of 1.96).
- *p*-value: .145, indicating a lack of statistical significance.
- Interpretation: While important, IC skills were not shown to have a significant direct influence on TI. This suggests that, while communication is a vital aspect of nursing, it may not be sufficient on its own to mitigate job-rotation-related turnover risk.

### Path Analysis Using Partial Least Squares–Structural Equation Modeling

The results of the PLS-SEM analysis offered valuable insights into the factors influencing TIs:

- Direct lineage to turnover intention (DL → TI): The path coefficient of .398 (*t*-value = 5.54, *p* < .001) indicates a significant positive correlation between unclear career pathways and increased turnover risk.
- Emotional response to turnover intention (ER → TI): The path coefficient of .239 (*t*-value = 2.52, *p* = .0116) highlights the importance of managing emotional stress during the rotation process.
- Job satisfaction to turnover intention (JS → TI): The negative correlation (−.264; *t*-value = 3.14, *p* = .0017) suggests that enhancing job satisfaction may help reduce turnover risk.

### Path Analysis (Supplementary)

The results of the PLS-SEM model (Figure 1) revealed significant and nonsignificant relationships between the constructs associated with the job rotation experience and turnover intention.

- DL → TI:
  The standardized path coefficient was .398 (*t*-value = 5.54, *p* < .001), indicating that a greater impact on daily life was associated with higher turnover intention.
- ER → TI:
  The standardized path coefficient was .239 (*t*-value = 2.52, *p* = .012), indicating that greater emotional strain during the rotation process was associated with higher turnover intention.
- JS → TI:
  The standardized path coefficient was −.264 (*t*-value = 3.14, *p* = .002), indicating that higher job satisfaction was associated with lower turnover intention.
- IC → TI:

This relationship was not statistically significant (*t*-value = 1.46, *p* = .145). Therefore, no direct significant association between interpersonal communication and turnover intention was observed.

Other significant paths included the effects of perceptions of the rotation process (RP) on job performance post-rotation (JP), job role behavior (RB), and job satisfaction during rotation (JS; all *p* < .001). Resilience during rotation (RR) was significantly associated with emotional response (ER), interpersonal communication (IC), and impact on daily life (DL; *p* = .012, .016, and .016, respectively).

### Analysis of the Feedback From the Open-ended Responses

The open-ended responses were categorized into five primary themes (Table 1).

**Table 1:** Breakdown of Participants’ Main Concerns (*N* = 246)

Category	*n*	%
1. Transparency of rotation preferences and assignment	86	35
2. Adaptation and support mechanisms	62	25
3. Rotation timing and flexibility	49	20
4. Individual preferences and choices	37	15
5. Work environment and culture	12	5

### Analysis of Participant Feedback

Participant feedback was classified into themes reflecting positive and negative sentiments toward the current system. The themes are shown in Table 2.

**Table 2:** Participant Perceptions of the Rotation System

Category	Main Suggestion	Proportion (%)
VS	Voluntarily rotate and respect individual preferences and choices.	45
AI	Address stress due to new environments to minimize turnover.	25
NMT	Extend training; provide better support systems and training materials.	15
RPA	Assign rotations based on preferences, with options to return to previous units.	20
RF	Extend the rotation cycle or adjust the frequency based on individual adaptation levels	10
ERS	Abolish the rotation system; it increases stress without enhancing nursing quality.	5

*Note.* VS=voluntary system; AI=adaptation issues; NMT=need for more training; RPA=rotation preferences and allocation; RF=rotation frequency; ERS=eliminating the rotation system.

### Categorization of Feedback: Positive and Negative Perspectives

Most of the participants (148; 60.2%) provided constructive suggestions. The feedback was divided into positive and negative perspectives:

### Positive Feedback (40%)

Participants who expressed positive perspectives toward the rotation system primarily emphasized flexibility, supportive adaptation mechanisms, and transparent allocation processes. Common suggestions included the following: (1) Implement a voluntary rotation system, (2) Provide adequate training and sufficient adaptation time, (3) Ensure transparent and fair allocation of rotation units based on preferences, (4) Offer support for rotations when staffing is adequate, (5) Extend the rotation cycle to reduce frequency, and (6) Strengthen support and guidance post-rotation.

### Negative Feedback (60%)

Participants expressing negative perspectives mainly described emotional stress, inadequate adaptation support, and concerns regarding frequent rotations and career uncertainty. The major concerns included the following: (1) Strong opposition to mandatory rotations, (2) Psychological stress and adaptation challenges associated with rotations, (3) Increased turnover due to poor post-rotation adaptation, (4) Providing insufficient clinical training and guidance during the rotation process, (5) Perceived rotations occur rapidly, preventing the mastery of specialized knowledge, and (6) Dissatisfaction with rotation assignments not aligned with personal career goals.

### Key Negative Statements From Participants

The feedback from the participants revealed recurring negative themes related to the rotation system:

1. Unnecessary: “Everyone quits because of the rotation, and it doesn’t help with staffing. Readapting to a new unit takes 3–6 months, which may lead to resignation.”
2. Too frequent: “From a newcomer’s perspective, the two-year rotation comes too fast. It feels like, just as you get familiar with everything, you rotate and have to start over again.”
3. Added pressure: “The pressure from the rotation is overwhelming!” There are many initial difficulties, and it is uncertain whether the assigned unit will meet expectations.
4. Limited transfer flexibility: “If someone cannot adapt to the new unit post-rotation, then the period available to apply for a transfer should be shortened from one year to 3–6 months to reduce turnover due to poor adaptation.”
5. Stress from rotation: “Rotation is stressful. Just when you’re comfortable in your current unit, you are moved to an unfamiliar environment.”
6. Lack of support: “Three days of rotation training is too short; it feels rushed.”
7. Opposition to mandatory rotation: “The mandatory nature of rotations is too forceful.”
8. Mismatch with preferences: “Rotation preferences are often not respected. It is said to broaden professional knowledge, but it seems aimed at filling staff shortages. The process lacks transparency.”

### Key Positive Suggestions From Participants

The participants also shared positive suggestions for improving the rotation system:

1. Voluntary system: Encourage voluntary participation in rotations based on individual development needs.
2. More training and support: Offer more comprehensive training resources, including manuals and online courses, and extend clinical training and orientation support during rotations
3. Preference-based assignments: Increase transparency in assignments; ensure assignments align with individual preferences; and offer alternatives if an initial assignment does not match expectations.
4. Extended adaptation period: Implement a longer post-rotation adaptation period with adequate emotional support and mentorship.
5. Flexible rotation timing: Consider extending the rotation cycle to 3–4 years or adjusting the frequency based on each nurse’s adaptation level.
6. Diverse departmental choices: Provide nurses with a wider range of specialties to choose from to facilitate career growth aligned with their interests.

### Strengths of the Rotation System

While most feedback was critical of the system, some highlighted its benefits and how the system might be improved, particularly in the area of professional development and skill acquisition (Table 3).

**Table 3:** Participant Perceptions of Rotation System Strengths

Category	Description	Proportion of Responses (%)
GEK	Enables learning of departmental routines, enhancing the ability to handle diverse medical conditions.	30
IWE	Provides opportunities to move from unfavorable work environments, potentially improving team dynamics.	20
PPG	Encourages the acquisition of new skills, fostering professional and personal development.	25
ERE	Elevates familiarity with routines across departments, helping in emergency response preparedness.	10
RWB	Provides a change in the environment, mitigating workplace fatigue and maintaining engagement.	5
ICF	Facilitates the exploration of different specialties, aiding in identifying suitable career paths.	5
RDT	Cross-departmental exposure enhances multidisciplinary cooperation skills.	5

*Note.* GEK=gaining experience and knowledge; IWE=improving work environment; PPG=promoting personal growth; ERE=enhancing response to emergencies; RWB=reducing workplace burnout; ICF=increasing career flexibility; RDT=reinforcing multidisciplinary teamwork.

### Recommendations Based on the Open-Ended Feedback

The open-ended feedback provided valuable insights for improving the rotation system:

1. Implement a more transparent and inclusive rotation planning process: Develop a system that allows nurses to express their preferences, with transparent criteria used in rotation assignments.
2. Enhance support systems: Introduce comprehensive training programs and mentorship initiatives to support adaptation to new units.
3. Review rotation frequency and necessity: Consider reducing the frequency of mandatory rotations or offering them as optional based on staff feedback and departmental needs.
4. Customize rotations to individual skills and career goals: Align rotations with the professional aspirations of nurses to foster career growth and job satisfaction.
5. Promote a better feedback mechanism: Regularly gather and analyze feedback to identify areas for improvement and respond in a timely manner to the concerns of nurses.

### Affect Valuation Theory Analysis

AVT provided the framework in this study for understanding the discrepancy between actual emotional experiences and ideal emotional states. AVT was used to evaluate the emotional responses of the participants following job rotations, highlighting the impact of these responses on psychological well-being and job performance. The results revealed a gap between ideal and actual emotional states, underscoring a need for more effective emotional management and support systems to ensure job rotations contribute positively to both professional and psychological outcomes.

### Positive Emotions: Enthusiasm, Excitement

Positive emotions such as enthusiasm, excitement, and happiness were compared to actual emotional responses to ideal states. The data suggest the positive emotions during the rotations were significantly lower than the ideal, indicating unmet emotional expectations.

#### Enthusiasm

- Actual mean=2.59, median=2 | ideal mean=3.24, median=3.
- Analysis: Enthusiasm levels were found to be considerably lower than their ideal state, reflecting a lack of motivation and drive during the rotation process. The demanding nature and heavy workloads associated with clinical work may have contributed to this diminished enthusiasm.

#### Excitement

- Actual mean=2.03, median=2 | ideal mean=3.18, median=3.
- Analysis: Excitement levels were found even further below ideal, indicating job rotations lack perceived novelty and challenge, which may decrease engagement and increase risk of burnout.

### Negative Emotions: Stress, Fatigue, and Anxiety

The data highlighted that negative emotions, including stress, fatigue, and anxiety, were significantly higher than their ideal states in this study, highlighting the psychological strain caused by rotations.

#### Stress

- Actual mean=3.09, median=3 | ideal mean=1.74, median=2.
- Analysis: Post-rotation stress levels were identified as notably higher than ideal, suggesting the rotation system imposes a substantial psychological burden. The need to adapt to new environments, increased responsibilities, and challenges in team integration are factors known to elevate stress.

#### Fatigue

- Actual mean=3.61, median=4 | ideal mean=2.24, median=2.
- Analysis: Fatigue was the most pronounced emotional reaction identified, with levels significantly exceeding the ideal. Factors such as long working hours, irregular schedules, and high workloads during the rotation process were likely contributors to the physical and emotional exhaustion observed.

#### Anxiety

- Actual mean=2.75, median=2.5 | ideal mean=1.52, median=1.
- Analysis: Anxiety levels were notably higher than the ideal, reflecting elevated feelings of uncertainty and stress, particularly in the face of new environments and unfamiliar tasks. Elevated anxiety is a factor known to negatively impact job satisfaction and work performance.

### Neutral Emotions: Calmness and Loneliness

The assessment of neutral emotions generated mixed results. While some participants were able to maintain composure under stress, others reported feelings of isolation due to frequent departmental shifts.

#### Calmness

- Actual mean=2.88, median=3 | ideal mean=2.71, median=3.
- Analysis: Calmness registered a slightly higher level than ideal, suggesting the participants were able to maintain emotional stability in high-pressure situations and/or were emotionally desensitized to stress.

#### Loneliness

- Actual mean=1.85, median=2 | ideal mean=1.74, median=2.
- Analysis: Sense of loneliness in this study approximated the ideal, suggesting that some of the participants received adequate support from their colleagues, even though frequent rotations made it challenging to build deep within-team connections.

### Gap Between Ideal and Actual Experiences

The results of the AVT analysis revealed a significant gap between actual and ideal emotional states, particularly in terms of positive and negative emotions (Table 4). These gaps underline the effects of the emotional strain associated with the current job rotation system, suggesting that efforts to enhance clinical skills must be accompanied by sufficiently robust emotional and psychological support mechanisms.

**Table 4:** Comparison of Actual and Ideal Emotional States During the Rotation Process

Emotional Aspect	Actual Mean	Actual Median	Ideal Mean	Ideal Median	Emotional Gap
Enthusiasm	2.59	2	3.24	3	−0.65
Excitement	2.03	2	3.18	3	−1.15
Stress	3.09	3	1.74	2	+1.35
Fatigue	3.61	4	2.24	2	+1.37
Anxiety	2.75	2.5	1.52	1	+1.23
Calmness	2.88	3	2.71	3	+0.17
Loneliness	1.85	2	1.74	2	+0.11

## Discussion

The findings of this study highlight a complex paradox within the nursing job rotation system. While cultivating adaptable, skilled professionals, rotation systems also introduce emotional and psychological stressors known to increase turnover intention (Kim & Han, 2020). This dual effect aligns with Conservation of Resources, which posits that individuals strive to preserve physical and emotional resources. When rotation systems do not provide adequate support and flexibility, these resources may be depleted, leading to burnout, dissatisfaction, and an increased desire to leave (Bae, 2022; Xu et al., 2023).

Under AVT, PGY nurses experience a significant gap between actual and ideal emotional states during the rotation process (Tsai, 2007). Positive emotions such as enthusiasm and excitement consistently fall below expectations, while negative emotions such as stress, fatigue, and anxiety, in particular, are elevated. These emotional discrepancies suggest the current rotation system may fail to meet the psychological needs of nurses, undermining their emotional well-being and job performance (Kim & Han, 2020).

However, factors that mitigate these negative effects and bolster job satisfaction were also identified in this study. Clear career pathways, structured support systems, and professional development opportunities may ease the emotional burden of rotations (Dong et al., 2023). The negative correlation observed between job satisfaction and turnover intention reinforces the importance of improving work environments and aligning rotations with career goals to enhance retention (Bracarense et al., 2022).

Exploring alternative strategies such as elective or flexible rotation policies and data-driven staffing solutions may better align institutional needs with individual nurse preferences. Moreover, these strategies may be a part of a customized professional development pathway supported by principles of magnet hospitals and positive organizational culture (Hegazy et al., 2021), which have become particularly crucial in the post-pandemic era, a period in which human resource resilience and retention are global nursing priorities.

### Practical Implications

Based on the findings, several pragmatic strategies are proposed to address the identified challenges and optimize job rotation practices.

1. *Flexible rotation policies*
  One-size-fits-all approaches are often ineffective due to the diverse needs and career goals of nursing staff. Introducing flexibility by, for example, allowing voluntary participation, longer adaptation periods, and/or the alignment of rotations with individual preferences may be used to help reduce stress and improve emotional well-being. Customization may be expected to encourage greater engagement and support long-term retention.
2. *Enhanced support systems*
  The findings underscore the need for structured support during the rotation process. Comprehensive training programs, mentorship, and emotional support may be used to ease transitions. Pre-rotation preparation should include emotional regulation and stress management training to equip nurses for the psychological demands of their new environment.
3. *Transparent career development*
  Clear communication regarding rotation criteria, career advancement opportunities, and professional development pathways may be emphasized to reduce uncertainty. Transparent decision-making for unit assignments and incorporating the rotation preferences of the affected nurses can alleviate anxiety and promote job satisfaction.
4. *Balanced workloads and adequate rest periods*

Given the high levels of reported stress and fatigue, adjusting workloads is critical. Introducing rest periods between rotations, allowing sufficient recovery time, and avoiding back-to-back transitions may be used to reduce fatigue, promoting both physical recovery and psychological resilience to enable nurses to maintain a positive and proactive work attitude.

### Integrating Emotional Support and Team Dynamics

A significant aspect of job rotation optimization encompasses addressing the emotional challenges revealed by the AVT analysis in this study. Comprehensive emotional support is crucial to narrowing the gap between actual and ideal emotional states, as detailed below:

#### Emotional counseling and psychological support

Access to counseling services and regular emotional health assessments should be incorporated into rotation protocols to provide nurses a platform to express concerns and obtain professional guidance to mitigate anxiety and foster emotional resilience.

#### *Team* *building and interpersonal communicati* *on*

Frequent departmental changes can disrupt a sense of belonging in nurses, leading to feelings of isolation. Enhancing team cohesion using regular team-building activities, mentorship, and peer support systems may be used to help mitigate these effects, strengthen interpersonal bonds, and elevate overall JS.

### Addressing the Affect Valuation Theory Gap

The AVT results demonstrated that the actual emotional states of nurses during the rotation process often fall short of their expectations, especially in the realm of positive emotions. Thus, institutions should focus on strategies that promote positive emotions and mitigate negative emotional reactions.

#### Career development and recognition

Expanding opportunities for career advancement, skill development, and formal recognition may be used to help enhance positive emotions such as enthusiasm and motivation, helping better align actual experiences with ideal emotional states and fostering greater engagement during the rotation process.

#### Varied and stimulating work environments

Encouraging cross-departmental collaboration and offering diverse, meaningful tasks may be employed to create a more dynamic and fulfilling work environment. Also, involving nurses in specialized projects or leadership roles during the rotation process may help promote professional growth and reduce emotional fatigue.

### Limitations and Future Research

This study was affected by several limitations. First, the cross-sectional design used restricts the ability to assess the long-term outcomes of job rotations. Nurse maturation, i.e., the progression of clinical skills, professional identity, and emotional resilience with experience, is known to significantly shape perceptions of job rotations. Highly experienced nurses generally handle stress better, demonstrate higher levels of professional adaptability, and integrate more effectively into team-based settings than their less-experienced peers. These factors may moderate rotation-related turnover intention (Ojala et al., 2025; Rees et al., 2022). A longitudinal approach should be adopted in future research to track how emotional and professional responses evolve over time to provide deeper insights into the sustained impact of well-managed rotations. Second, the scope of emotional variables considered in this study was limited. While the AVT provided a robust framework, key factors such as organizational culture, social support, and personal coping strategies were not fully examined. Incorporating these and other relevant factors in future studies may be expected to provide a more comprehensive understanding of the emotional dynamics influencing nurse retention. Third, the generalizability of the findings is limited by the relatively small sample size, homogenous population, and single-institution setting. Broader, multi-site studies with more diverse samples are needed to strengthen the generalizability of findings across diverse healthcare contexts. In addition, several nonsignificant pathways in the PLS-SEM model suggest that other unmeasured mediating variables may influence turnover intention and should be explored in future studies. Finally, this study was conducted during the COVID-19 pandemic, a period marked by frequent and often urgent staffing reallocations. Many nurses were assigned to different departments due to infection control needs, isolation requirements, confirmed cases, and flexible workforce deployment strategies. Consequently, several staff members were rotated not as part of planned professional development, but rather due to necessity.

Interestingly, the qualitative follow-up collected using open-ended survey responses and informal interviews revealed that several participants, after experiencing unexpected rotations, acknowledged and accepted management’s stated rationale of the rotation system being important to building a versatile workforce. These participants reported more favorable attitudes toward rotation, especially when they recognized its value in broadening their competencies and improving team dynamics. Although testing workforce flexibility/resilience cannot wait for the next pandemic, these insights underscore the potential of fostering a proactive and growth-oriented mindset toward rotation that encourages nurses to step outside their comfort zones for long-term professional development (Farokhzadian et al., 2024; Labrague & de los Santos, 2021; Shahrbabaki et al., 2023).

### Conclusions

The results of this research highlight the dual nature of job rotation systems within the nursing profession. While job rotation is a valuable tool for professional development, it poses significant emotional and psychological challenges to those affected.

By adopting a human-centered approach to job rotation, healthcare institutions may proactively create a more supportive work environment, better retain skilled nursing professionals, and elevate the quality of patient care. Fostering professional identity and job satisfaction is crucial to reducing turnover intention among nurses, and incorporating tailored support systems, clear career pathways, and well-being initiatives is essential to the success of rotation programs. Further research into the long-term and contextual factors affecting job rotations is necessary to provide deeper insights into best practices and, ultimately, realize a more positive and effective healthcare system.
